# Lipopolysaccharide Modifies Sodium Current Kinetics through ROS and PKC Signalling in Induced Pluripotent Stem-Derived Cardiomyocytes from Brugada Syndrome Patient

**DOI:** 10.3390/jcdd9040119

**Published:** 2022-04-15

**Authors:** Zhenxing Liao, Yingrui Li, Xuehui Fan, Zhen Yang, Ibrahim El-Battrawy, Xiaobo Zhou, Ibrahim Akin

**Affiliations:** 1 Department of Cardiology, Angiology, Haemostaseology and Medical Intensive Care, Medical Faculty Mannheim, University Medical Centre Mannheim (UMM), Heidelberg University, 68167 Mannheim, Germany; liao.zhenxing@medma.uni-heidelberg.de (Z.L.); yingrui.li@medma.uni-heidelberg.de (Y.L.); xuehui.fan@medma.uni-heidelberg.de (X.F.); zhen.yang@medma.uni-heidelberg.de (Z.Y.); ibrahim.elbattrawy2006@gmail.com (I.E.-B.); ibrahim.akin@umm.de (I.A.); 2 Department of Thoracic Surgery, Affiliated Hospital of North Sichuan Medical College, Nanchong 637000, China; 3 Key Laboratory of Medical Electrophysiology of Ministry of Education and Medical Electrophysiological Key Laboratory of Sichuan Province, Collaborative Innovation Center for Prevention of Cardiovascular Diseases, Institute of Cardiovascular Research, Southwest Medical University, Luzhou 646000, China; 4 European Center for AngioScience (ECAS) and German Center for Cardiovascular Research (DZHK) Partner Site Heidelberg/Mannheim, 68167 Mannheim, Germany

**Keywords:** inflammation, Brugada syndrome, sodium channel, Lipopolysaccharide, human-induced pluripotent stem cell-derived cardiomyocyte

## Abstract

Studies have suggested a connection between inflammation and arrhythmogenesis of Brugada syndrome (BrS). However, experimental studies regarding the roles of inflammation in the arrhythmogenesis of BrS and its underlying mechanism are still lacking. This study aimed to investigate the influence of inflammation on BrS-phenotype features using human-induced stem cell-derived cardiomyocytes (hiPSC-CMs) from a BrS-patient carrying an SCN10A variant (c.3749G > A). After LPS treatment, the peak sodium current decreased significantly in SCN10A-hiPSC-CMs, but not in healthy donor-hiPSC-CMs. LPS also changed sodium channel gating kinetics, including activation, inactivation, and recovery from inactivation. NAC (*N*-acetyl-l-cysteine), a blocker of ROS (reactive oxygen species), failed to affect the sodium current, but prevented the LPS-induced reduction of sodium channel currents and changes in gating kinetics, suggesting a contribution of ROS to the LPS effects. Hydrogen peroxide (H_2_O_2_), a main form of ROS in cells, mimicked the LPS effects on sodium channel currents and gating kinetics, implying that ROS might mediate LPS-effects on sodium channels. The effects of H_2_O_2_ could be attenuated by a PKC blocker chelerythrine, indicating that PKC is a downstream factor of ROS. This study demonstrated that LPS can exacerbate the loss-of-function of sodium channels in BrS cells. Inflammation may play an important role in the pathogenesis of BrS.

## 1. Introduction

The Brugada syndrome (BrS) is a genetic life-threatening channelopathy. The electrocardiogram (ECG) of the patient is characterized by an elevated ST segment in the left precordial leads V1–V3 and right bundle branch block. The incidence of BrS in males is higher than that in females and is the highest in Southeast Asia [1,2]. Arrhythmias of BrS appear often at rest or during sleep. Aside from accidents, BrS caused the most death cases in men < 40 years old [3].

The first gene that was linked to BrS was the cardiac sodium channel SCN5A gene [4]. In the past two decades, hundreds of mutations or variants in 43 genes have been identified to be possible pathogenic factors [5]. Among those, SCN5A which encodes the α subunit of the cardiac sodium channel (Nav1.5) was most frequently detected in BrS-patients [6]. Mutations in β subunit genes of the Nav1.5 sodium channels, such as SCN1B, SCN2B, and SCN3B, were also identified in some BrS-patients. Mutations in the SCN10A gene that encodes the Nav1.8 sodium channel, were detected in BrS-patients and were demonstrated to reduce peak sodium current (I_Na_) in the human-induced pluripotent stem cell-derived cardiomyocytes derived from a BrS-patient [7]. In addition to ion channel genes, other genes, such as the RAN guanine nucleotide release factor (RANGRF) gene [8,9], the glycerol-3-phosphate dehydrogenase 1-like gene (GPD1L) [10], and the gene for Plakophilin-2 (PKP2) [11] can influence the expression or trafficking of the sodium channel. Mutations in those genes can cause a reduction of I_Na_ and thus lead to BrS. Furthermore, besides sodium channels, a dysfunction of calcium channels and potassium channels may also contribute to the pathogenesis of BrS. Mutations in L-type calcium channels were reported to be associated with an overlap of BrS and short QT syndrome [12]. Moreover, potassium channel mutations were linked to BrS, too. Gain of function mutations in KCNE3 and KCNE5, which can enhance transient outward potassium channel currents (I_to_), were described as possible contributors to the pathogenesis of BrS [13,14].

It is widely believed that BrS is caused by the dysfunction (either loss or gain of function) of some ion channels or their regulating molecules, resulting in abnormal cardiac action potential (AP) or the disruption of conduction. These electrical abnormalities can increase the susceptibility to the occurrence of arrhythmias or even SCD, typically in the absence of a structural abnormality of the heart [15]. However, accumulating evidence indicated that factors besides genetic mutations may cause arrhythmias via altering ion channel function. In addition to a well-recognized list of drugs directly interfering with cardiac ion channel function, in recent years, several studies suggested that immunologic and inflammatory factors play an important role in the arrhythmogenesis of the disorder [16]. For example, with autoantibodies, such as anti-hERG channel antibodies (anti-Ro/SSA), anti-Kv1.4 channel antibodies can inhibit the hERG (I_Kr_) channel and I_to_ channels, respectively, while anti-Kv7.1channel antibodies can activate I_Ks_ channels [17]. In addition, some cytokines, such as TNF-α and IL-6, were shown to inhibit I_Kr_, I_Ks,_ or I_to_, while IL-6 was shown to inhibit I_Kr_, and IL-1 was shown to inhibit I_to_ [17]. IL-2 can increase SCN3B sodium channel expression and current [18]. TNF-α can regulate Nav1.8 sodium channels via p38-MAPK, JNK, and ERK pathways [19]. However, to date, these factors have been largely overlooked in the field despite they probably contributed to some unexplained arrhythmias/SCD. 

Fever, a hallmark of infection and inflammatory disease, is a well-known trigger for the occurrence of arrhythmias in BrS [20]. The febrile temperature is so closely associated with the intensity of the inflammatory response that inflammation seems to be the real “culprit” in fever-induced BrS. Indeed, the evidence tends to suggest that inflammation may be involved in the pathogenesis of BrS. Frustaci et al. reported histologic evidence of fibrosis, myocarditis, and inflammatory infiltrates in BrS-patients [21]. Another study reported that C-reactive protein (CRP) levels >2 mg/L are an independent marker for being symptomatic [22]. Anthony Li reported two BrS-patients showing a connection between acute inflammation and episodes of ventricular fibrillation [23]. Furthermore, it was shown that a cocktail of inflammatory mediators containing bradykinin, PGE-2, histamine, 5-HT, and adenosine 5′-triphosphate altered the gating properties of Nav1.5 and both protein kinase A and protein kinase C mediated the inflammatory mediators induced alteration in the gating properties of Nav1.5 [24]. Taken together, the findings suggested that inflammation may participate in the disease process of BrS. However, experimental study on the influences of inflammation on BrS is still lacking. Whether inflammation or inflammatory factors contribute to the arrhythmogenesis of BrS is not clear. More importantly, whether the mutation in the sodium channel alters the effects of inflammatory factors or their relating signaling on the channel gating has not been investigated.

Since fever can trigger the occurrence of arrhythmias in BrS and is usually caused by infections, we hypothesize that the inflammation may enhance the loss-of-function of the sodium channel in BrS-cardiomyocytes. Therefore, we designed this study to investigate possible roles and mechanisms of LPS on peak sodium channel currents in human-induced pluripotent stem cell-derived cardiomyocytes from a patient with BrS.

## 2. Materials and Methods

### 2.1. Ethics Statement

Skin biopsies were obtained with written informed consent from three healthy donors and a BrS-patient. The Ethical Committee of the Medical Faculty Mannheim, University of Heidelberg (approval number: 2009-350N-MA) approved the study. The study was performed according to the approved guidelines and conducted in accordance with the Helsinki Declaration of 1975, as revised in 1983.

### 2.2. Generation of Human iPS Cells

The human iPSC lines from three healthy donors and a BrS-patient carrying an SCN10A variant (c.3749G > A) used in this study were provided by Dr. Cyganek’s group (the Stem Cell Unit, Clinic for Cardiology and Pneumology, University Medical Center Göttingen, Göttingen, Germany). A detailed description of the generation of the hiPS cell lines has been provided in our recent publications [7,25,26,27]. 

### 2.3. Generation of hiPSC-CMs

The hiPSCs were cultured without feeder cells and differentiated into cardiomyocytes (hiPSC-CMs) as reported previously with some changes [27]. Culture flasks were coated with Matrigel (Corning, NY, USA). The medium for hiPSC-CM culture in RPMI 1640 Glutamax (Life Technologies, Waltham, MA, USA) contained penicillin/streptomycin, sodium pyruvate, ascorbic acid (Sigma Aldrich, Taufkirchen, Germany), and B27 (Life Technologies). After three times of passaging, hiPSC colonies were transferred to feeder-free 6-well plates and cultured with TeSR-E8 for expansion. When cells reached 85–95% confluence, cardiomyocyte differentiation was started. CHIR99021 (Stemgent, Cambridge, MA, USA) and IWP-4 (Stemgent, Cambridge, MA, USA) were added at different time points to induce iPS cells to differentiate into cardiomyocytes (hiPSC-CMs). During the first 2 weeks after the onset of differentiation, beating cell colonies could be seen on the 6th day or later. In the third week, a selection medium containing lactate (Sigma, Taufkirchen, Germany) and RPMI medium without glucose and glutamine (WKS, Germany) was applied for selecting the cardiomyocytes. From 30 days on, the hiPSC-CMs were cultured with a basic culture medium. For patch-clamp measurements, the hiPSC-CMs were dissociated from 6-well plates by 0.05% Trypsin-EDTA and plated on Matrigel-coated 3.5 cm Petri dishes as single cells. 

### 2.4. ROS Detection

Intracellular reactive oxygen species (ROS) generation was measured by *2′,7′-Dichlorofluorescin diacetate* (DCFH-DA). DCFH-DA is a cell-permeable non-fluorescent probe that can be oxidized to fluorescent *2′,7′-dichlorofluorescein* (DCF, Taufkirchen, Germany) by ROS. Cells were treated with LPS for 48 h. Then, they were washed two times and incubated with 5 μM of DCFH-DA solution in a serum-free medium at 37 °C for 30 min in the dark. A fluorescence microscope (BX51; Olympus Corp., Hamburg, Germany) was used to evaluate the DCF fluorescence of hiPSC-CMs in the dish.

### 2.5. Patch-Clamp

Whole-cell patch-clamp recording techniques were used to measure the peak sodium current (peak I_Na_). Borosilicate glass capillaries (MTW 150 F; world Precision Instruments, Inc., Sarasota, FL, USA) were pulled to form patch electrodes by using a DMZ-Universal Puller (Zeitz-Instrumente Vertriebs GmbH, Martinsried, Germany) and filled with pre-filtered pipette solution (see below). Current recordings were carried out at room temperature with an EPC-7 amplifier (HEKA Elektronik, Reutlingen, Germany), connected via a 16-bit A/D interface to a Pentium IBM clone computer. The signals were low-pass filtered (1 kHz) before 5 kHz digitization. Data acquisition and analysis were performed with an ISO-3 multitasking patch-clamp program (MFK M. Friedrich, Niedernhausen, Germany). 

Patch pipette resistances ranged from 1–2  MΩ. The electrode offset potential was zero-adjusted after the patch pipette was moved into the bath solution. After the patch pipette was carefully moved on the cell membrane by a micromanipulator, a slight suction (negative pressure) was given to establish a high-resistance (Giga-Ohm) seal between the cell membrane and the pipette wall (also called Giga-seal). When a Giga-seal was obtained, a fast capacitance current was compensated. Next, the membrane in the pipette tip was disrupted by suction for establishing the whole-cell configuration. Then, membrane capacitance (Cm) and series resistance (Rs) were compensated (60–80%). To examine the rundown of recorded currents, we carefully observed the time-dependent change of the currents. Recordings were started when the currents reached a steady state, normally within 1 to 3 min.

The current-voltage (I–V) relationships were obtained by plotting the current density against respective voltages. Voltage-dependent activation was estimated from peak conductance.

*G*_Na_ = I_Na_/(*V*_m_ − *V*_rev_)

*D*_inf(V)_ = *G*_Na_/*G*_Na max_

where I_Na_ is the peak sodium current, *G*_Na_ is the peak conductance, *D*_inf(V)_ is the steady-state activation parameter, and *G*_Na max_ is the maximum value of *G*_Na_. *V*_rev_ is the reversal potential for Na^+^ current and is measured as the zero-current potential in the I–V relation. *V*_m_ is the membrane potential (testing potential). Activation and inactivation curves were fitted with the following Boltzmann equation:

*y* = 1/(1 + exp(−(*V* − *V*_0.5_)/*k*))

where *k* represents the slope factor, *V* represents the test potential, and *V*_0.5_ is the voltage at which the conductance was half-maximal. 

To measure the recovery from the inactivation of sodium channels, a double-pulse protocol with increasing time intervals was applied. The second pulse elicited peak I_Na_ was normalized to that elicited by the first pulse and plotted against the time intervals between the two pulses. Then, curves were fitted by a mono-exponential equation to obtain the time constant of recovery (Tau). 

The bath solution for the peak sodium current (I_Na_) measurements contained (mmol/L): 20 NaCl, 110 CsCl1 MgCl_2_, 1.8 CaCl_2_, 10 glucose, 10 HEPES, pH 7.4 (CsOH). Microelectrodes were filled with (mmol/L): 2 CaCl_2_, 135 CsCl, 3 MgATP, 5 EGTA, 2 TEA-Cl, 10 HEPES, 10 NaCl, pH 7.2 (CsOH). To block the I_Ca-L_, 0.01 mmol/L nifedipine was added in the bath solution shortly before the measurements. 

### 2.6. Drugs

H_2_O_2_ (Fisher Scientific, Schwerte, Germany) stock solution (0–25 M) was prepared by diluting 30% H_2_O_2_ with water. LPS (Lipopolysaccharides from *E. coli*, source strain ATCC 12740, serotype 0127: B8, gel filtrated, gamma-irradiated, cell culture tested, Sigma L 4516) was dissolved in water. Nifedipine, *N*-acetylcysteine (NAC), chelerythrine chloride, and 2′,7′-Dichlorofluorescin diacetate were dissolved in DMSO.

### 2.7. Statistics

Data are shown as mean  ±  SEM and were analyzed using Excel 2016 software (Microcal Software, Inc., Northampton, MA, USA) and InStat© (GraphPad, San Diego, CA, USA) as well as SigmaPlot 11.0 (Systat GmbH, Frankfurt, Germany). An unpaired Student’s *t*-test was used for comparisons of two independent groups with normal distribution. For parametric data of more than two groups, a one-way ANOVA with a Holm–Sidak post-test for multiple comparisons (all treated groups versus control). *p* <  0.05 (two-tailed) was considered significant. 

## 3. Results

### 3.1. LPS Reduced Peak I_Na_ in hiPSC-CMs Derived from BrS-Patients

**Figure 1:**
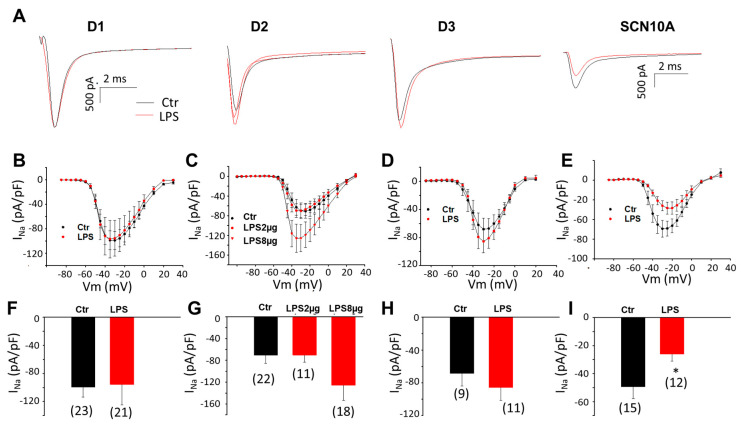
**LPS suppressed peak sodium channel currents in hiPSC-CMs from BrS-patients.** Cells were treated with vehicle (same amount of water, Ctr) or LPS of 2 µg/mL or 8 µg/mL (in D2 cells) for 48 h and then peak sodium channel currents (I_Na_) were recorded. (**A**) Representative traces of peak I_Na_ at −30 mV were recorded in hiPSC-CMs from healthy donors (D1, D2, and D3) and the BrS-patient (SCN10A) in absence (Ctr) and presence of LPS (2 µg/mL). (**B**) Current-Voltage (I–V) relationship curves of peak I_Na_ in hiPS-CMs from D1 donor in absence (Ctr) and presence of LPS (2 µg/mL). (**C**) Current-Voltage (I–V) relationship curves of peak I_Na_ in hiPS-CMs from D2 donor in absence (Ctr) and presence of LPS (2 µg/mL and 8 µg/mL). (**D**) Current-Voltage (I–V) relationship curves of peak I_Na_ in hiPS-CMs from D3 donor in absence (Ctr) and presence of LPS (2 µg/mL). (**E**) Current-Voltage (I–V) relationship curves of peak I_Na_ in hiPS-CMs from the BrS-patient (SCN10A) in absence (Ctr) and presence of LPS (2 µg/mL). (**F**) Mean values of peak I_Na_ at −30 mV in hiPS-CMs from D1 donor in absence (Ctr) and presence of LPS (2 µg/mL). (**G**) Mean values of peak I_Na_ at −30 mV in hiPS-CMs from D2 donor in absence (Ctr) and presence of LPS (2 µg/mL and 8 µg/mL). (**H**) Mean values of peak I_Na_ at −30 mV in hiPS-CMs from D3 donor in absence (Ctr) and presence of LPS (2 µg/mL). (**I**) Mean values of peak I_Na_ at −30 mV in hiPS-CMs from the BrS-patient in absence (Ctr) and presence of LPS (2 µg/mL). Numbers given in (**F**–**I**) represent the number of cells of measurements also for (B–E). * *p* < 0.05 according to *t*-test.

A recent study in our group demonstrated that hiPSC-CMs possess the molecular basis of inflammatory responses and could model inflammatory changes when they were challenged by LPS [28]. In addition, we found that the hiPSC-CMs from the patient carrying the variant (c.3749G > A) in SCN10A recapitulated the phenotypic feature (loss-of-function of cardiac sodium channel) of BrS. Therefore, we treated the SCN10A-hiPSC-CMs with LPS to mimic an inflammation to study the possible roles of inflammation on BrS phenotypic changes, focusing on changes in peak I_Na_. First, LPS of 2 µg/mL was applied for 48 h. In the control (donor) group, LPS treatment did not change the peak I_Na_ density significantly (Figure 1A–D,F–H). In SCN10A-hiPSC-CMs, LPS treatment induced a significant reduction of the peak I_Na_ (Figure 1A,E,I). Since 2 µg/mL of LPS failed to inhibit I_Na_ in donor-hiPSC-CMs, the LPS concentration was elevated to 8 µg/mL to examine its effect in D2-hiPSC-CMs. The peak sodium current density increased slightly in the presence of 8 µg/mL of LPS (Figure 1C,G).

**Figure 2:**
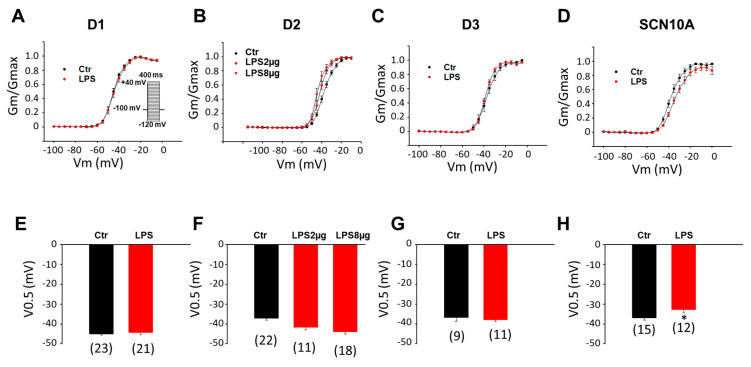
**LPS effects on activation of sodium channels in hiPSC-CMs from healthy donors and the BrS-patient.** Cells were treated with vehicle (same amount of water, Ctr) of LPS of 2 µg/mL or 8 µg/mL (in D2 cells) for 48 h. (**A**) Activation curves of peak sodium channel currents (I_Na_) recorded in hiPSC-CMs from D1 healthy donor. (**B**) Activation curves of peak I_Na_ recorded in hiPSC-CMs from D2 healthy donor. (**C**) Activation curves of peak I_Na_ recorded in hiPSC-CMs from D3 healthy donor. (**D**) Activation curves of peak sodium channel currents (I_Na_) recorded in SCN10A-hiPSC-CMs. (**E**) Mean values of potential at 50% activation (V0.5) in hiPSC-CMs from D1 healthy donor. (**F**) Mean values of potential at 50% activation (V0.5) in hiPSC-CMs from D2 healthy donor. (**G**) Mean values of potential at 50% activation (V0.5) in hiPSC-CMs from D3 healthy donor. (**H**) Mean values of potential at 50% activation (V0.5) in SCN10A-hiPSC-CMs. Numbers given in (**E**–**H**) represent the number of cells of measurements also for (**A**–**D**). * *p* < 0.05 versus Ctr according to *t*-test or one-way ANOVA with Holm-Sidak post-test (**F**).

To investigate the mechanisms underlying the reduction of peak I_Na_ caused by LPS, the Na channel gating kinetics, including activation, inactivation, and recovery from inactivation, were analyzed. In healthy (donor) hiPSC-CMs, LPS did not significantly change the activation curves in the D1 and D3 cell lines, although it slightly shifted the curves in D2 cells to more negative potentials (Figure 2A–C,E–G). In SCN10A-hiPSC-CMs, LPS attenuated the voltage-dependent activation of the Na channel by shifting the activation curve to more positive potentials (Figure 2D,H). 

**Figure 3:**
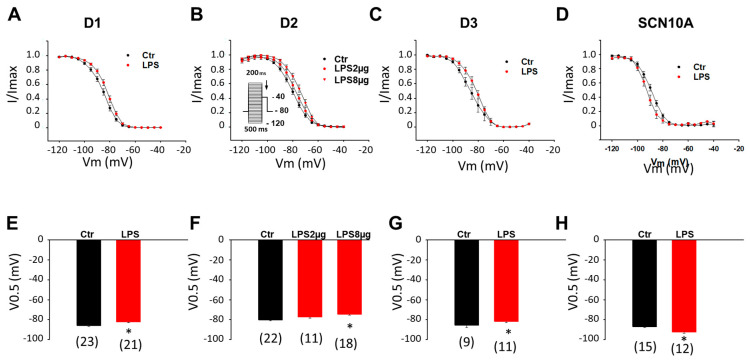
**LPS effects on inactivation of sodium channels in hiPSC-CMs from healthy donors and the BrS-patient.** Cells were treated with vehicle (Ctr) of LPS of 2 µg/mL or 8 µg/mL (in D2 cells) for 48 h. (**A**) Inactivation curves of peak sodium channel currents (I_Na_) recorded in hiPSC-CMs from D1 healthy donor. (**B**) Inactivation curves of peak I_Na_ recorded in hiPSC-CMs from D2 healthy donor. (**C**) Inactivation curves of peak I_Na_ recorded in hiPSC-CMs from D3 healthy donor. (**D**) Inactivation curves of peak sodium channel currents (I_Na_) recorded in SCN10A-hiPSC-CMs. (**E**) Mean values of potential at 50% inactivation (V0.5) in hiPSC-CMs from D1 healthy donor. (**F**) Mean values of potential at 50% inactivation (V0.5) in hiPSC-CMs from D2 healthy donor. (**G**) Mean values of potential at 50% inactivation (V0.5) in hiPSC-CMs from D3 healthy donor. (**H**) Mean values of potential at 50% inactivation (V0.5) in SCN10A-hiPSC-CMs. Numbers given in (**E**–**H**) represent the number of cells of measurements also for (**A**–**D**). * *p* < 0.05 versus Ctr according to *t*-test or one-way ANOVA with Holm-Sidak post-test (**F**).

To analyze the Na channel voltage-dependent inactivation, inactivation curves were obtained by plotting the relative Na currents (normalized to the maximal I_Na_) versus voltages. In all three donor cell lines, LPS reduced the voltage-dependent inaction by shifting the inactivation curves to more positive potentials (Figure 3A–C,E–G). In D2-hiPSC-CMs, a higher dose (8 µg/mL) was needed to obtain statistically significant effects (Figure 3B,F). In SCN10A-hiPSC-CMs, LPS enhanced the voltage-dependent inactivation and shifted the inactivation curves to more negative potentials (Figure 3D,H). 

**Figure 4:**
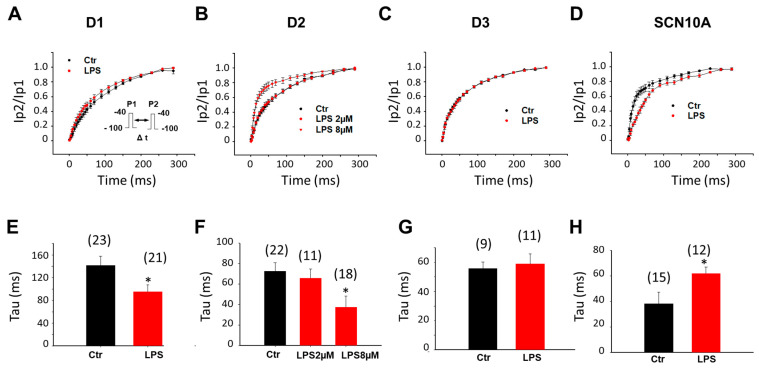
**LPS effects on recovery from inactivation of sodium channels in hiPSC-CMs from healthy donors and the BrS-patient.** Cells were treated with vehicle (Ctr) of LPS of 2 µg/mL or 8 µg/mL (in D2 cells) for 48 h. (**A**) Recovery curves of peak sodium channel currents (I_Na_) recorded in hiPSC-CMs from D1 healthy donor. (**B**) Recovery curves of peak I_Na_ recorded in hiPSC-CMs from D2 healthy donor. (**C**) Recovery curves of peak I_Na_ recorded in hiPSC-CMs from D3 healthy donor. (**D**) Recovery curves of peak sodium channel currents (I_Na_) recorded in SCN10A-hiPSC-CMs. (**E**) Mean values of time constant (Tau) of recovery from inactivation in hiPSC-CMs from D1 healthy donor. (**F**) Mean values of time constant (Tau) of recovery from inactivation in hiPSC-CMs from D2 healthy donor. (**G**) Mean values of time constant (Tau) of recovery from inactivation in hiPSC-CMs from D3 healthy donor. (**H**) Mean values of time constants (Tau) in SCN10A-hiPSC-CMs. Numbers given in (**E**–**H**) represent the number of cells of measurements also for (**A**–**D**). * *p* < 0.05 versus Ctr according to *t*-test or one-way ANOVA with Holm–Sidak post-test (**F**).

To analyze the time-dependent recovery of Na channels from inactivation, the recovery curves were obtained by plotting the recovered currents against the recovery time (the interval between two pulses). In D1-hiPSC-CMs, the time-dependent recovery was accelerated (smaller tau value) by LPS of 2 µg/mL (Figure 4A,E). In D2-hiPSC-CMs, the recovery was also accelerated, but at a higher concentration (8 µg/mL) of LPS (Figure 4B,F). In D3-hiPSC-CMs, the recovery was not changed (Figure 4C,G). In SCN10A-hiPSC-CMs, the time-dependent recovery was decelerated by LPS (Figure 4D–H). 

### 3.2. ROS and PKC Blocker Attenuated Effects of LPS on Peak I_Na_

**Figure 5:**
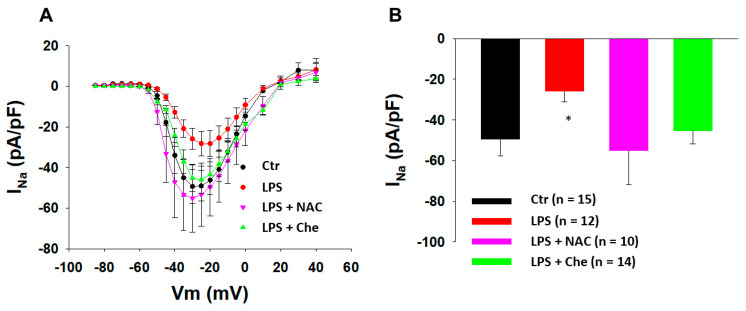
**A ROS and PKC blocker prevented the LPS effects on sodium channel currents in SCN10A-hiPSC-CMs.** Cells were treated for 48 h with vehicle (Ctr) or LPS of 2 µg/mL (LPS) or LPS plus 1 mM *N*-acetylcysteine (LPS + NAC) or LPS plus 5 µM chelerythrine (LPS + Che). (**A**) I–V curves of peak sodium channel currents (I_Na_) recorded in SCN10A-hiPSC-CMs. (**B**) Mean value of peak I_Na_ at −30 mV. Numbers given in (**B**) represent the number of cells of measurements also for (**A**). * *p* < 0.05 versus Ctr according to one-way ANOVA with Holm–Sidak post-test.

**Figure 6:**
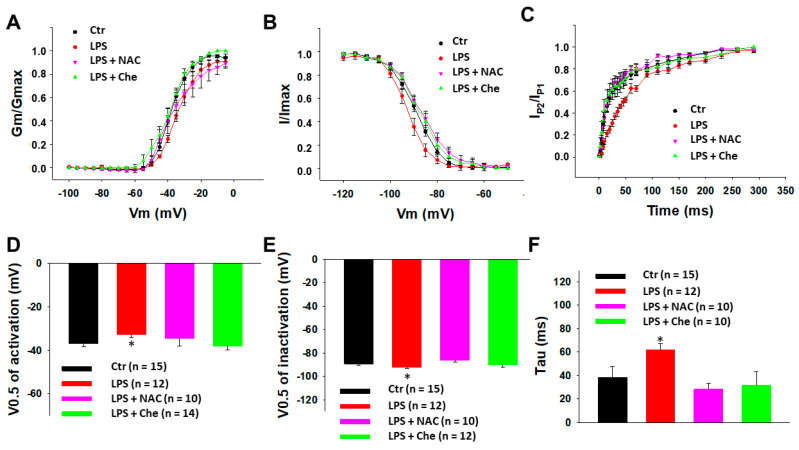
**A ROS and PKC blocker prevented the LPS effects on sodium channel gating kinetics in SCN10A-hiPSC-CMs.** Cells were treated for 48 h with vehicle (Ctr) or LPS of 2 µg/mL (LPS) or LPS plus 1 mM *N*-acetylcysteine (LPS + NAC) or LPS plus 5 µM chelerythrine (LPS + Che). (**A**) Activation curves of peak sodium channel currents (I_Na_) recorded in SCN10A-hiPSC-CMs. (**B**) Inactivation curves of peak I_Na_ recorded in SCN10A-hiPSC-CMs. (**C**) Recovery curves of peak I_Na_ recorded in SCN10A-hiPSC-CMs. (**D**) Mean values of V0.5 of activation. (**E**) Mean values of V0.5 of inactivation. (**F**) Mean values of time constants (Tau) of recovery from inactivation. Numbers given in (**D**–**F**) represent the number of cells of measurements also for (**A**–**C**). * *p* < 0.05 versus Ctr according to one-way ANOVA with Holm–Sidak post-test.

Studies reported that ROS contributed to the regulation of sodium currents in neurons and cardiomyocytes [29,30] and ROS signaling was involved in inflammation [31]. We suppose that ROS may be involved in the LPS-induced reduction of peak I_Na_ in the BrS-hiPSC-CMs. Hence, the SCN10A-hiPSC-CMs were treated for 48 h with LPS (2 µg/mL) or LPS plus *N*-acetylcysteine (NAC, 1 mM), a ROS scavenger. The result showed that peak I_Na_ was not changed by the NAC alone, but NAC prevented the inhibitory effect of LPS on peak I_Na_ (Figure 5). Likewise, NAC abolished the LPS effects on activation, inactivation, and the recovery of peak I_Na_ in SCN10A-hiPSC-CMs (Figure 6). These data suggested that ROS signaling mediated the inhibition of the Na channels by LPS. In addition to NAC, a PKC inhibitor (chelerythrine, 5 µM) also blocked the LPS effects (Figure 5 and Figure 6), indicating an involvement of PKC in the LPS effects on sodium channels.

**Figure 7:**
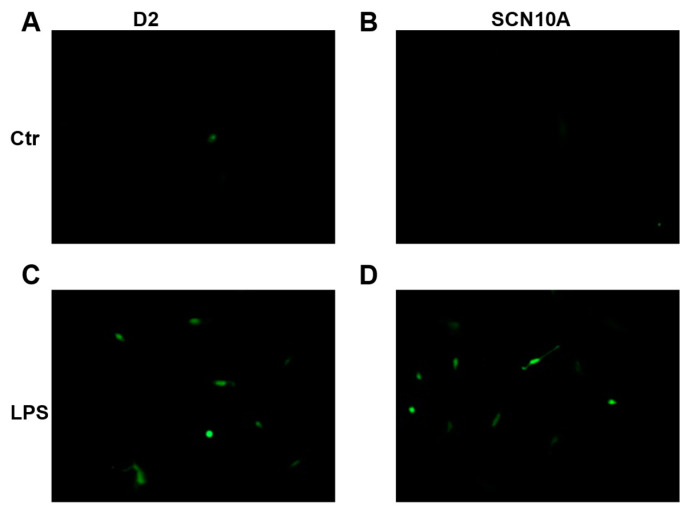
**LPS-induced ROS generation in the donor and BrS-hiPSC-CMs.** Cells from D2 donor (Ctr) and BrS-patient (SCN10A) were incubated with LPS (2 µg/mL) for 48 h. Next, hiPSC-CMs were washed twice and incubated with 5 μM DCFH-DA solution in serum-free medium at 37 °C for 30 min in the dark. A fluorescence microscope was employed for evaluating the DCF fluorescence of cells on coverslips. (**A**,**B**) *Fluorescence* intensity in control cells (Ctr) without LPS treatment. (**C**,**D**) *Fluorescence* in the LPS treated cardiomyocytes. Magnification, ×400.

LPS was found to cause tissue injury partially through ROS [31]. In cardiomyocytes, a low concentration of LPS (20 ng/mL) was shown to cause a significant increase in ROS [32]. Therefore, we assessed the ROS generation in donor- and SCN10A-hiPSC-CMs challenged by LPS. After hiPSC-CMs were treated with 2 μg/mL of LPS for 24 h, fluorescence imaging of 2,7-dichlorofluorescein (DCF) was applied to detect ROS generation as described previously [33]. DCF fluorescence of cells on coverslips showed that the fluorescence intensity was very weak without LPS treatment, but significantly intensified by the LPS treatment (Figure 7) in D2- and SCN10A-hiPSC-CMs, indicative of an elevation of ROS generation in donor- and BrS-hiPSC-CMs in the presence of LPS.

### 3.3. Peroxide Decreased the Peak I_Na_ in BrS-hiPSC-CMs

**Figure 8:**
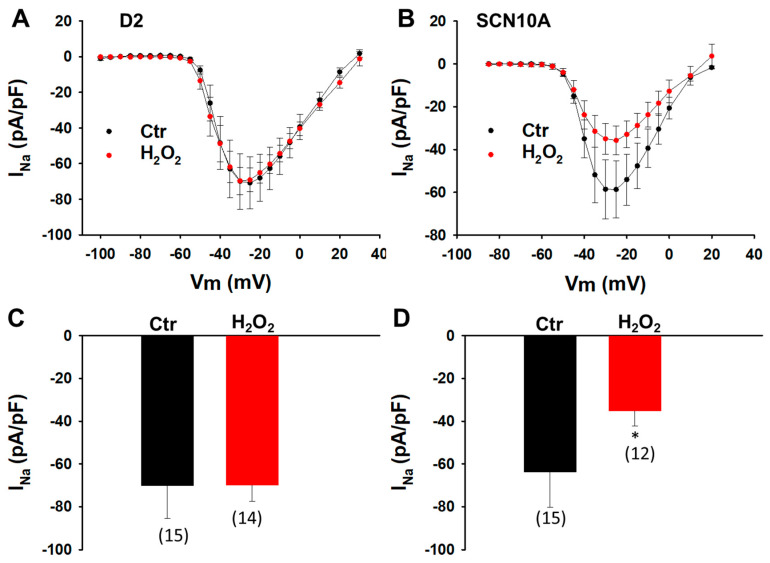
**Peroxide (H_2_O_2_) decreased the peak I_Na_ in SCN10A cardiomyocytes.** Cells were treated with vehicle (water, Ctr) or H_2_O_2_ (200 µM) for 2 h. (**A**) I–V curves of peak I_Na_ in donor-hiPSC-CMs (D2) in absence (Ctr) or presence (H_2_O_2_) of H_2_O_2_. (**B**) I–V curves of peak I_Na_ in SCN10A-hiPSC-CMs (SCN10A) in absence (Ctr) or presence (H_2_O_2_) of H_2_O_2_. (**C**) Mean values of peak I_Na_ at −30 mV in donor-hiPSC-CMs in absence (Ctr) or presence (H_2_O_2_) of H_2_O_2_. (**D**) Mean values of peak I_Na_ at −30 mV in SCN10A-hiPSC-CMs in absence (Ctr) or presence (H_2_O_2_) of H_2_O_2_. Values given are mean  ±  SEM. Numbers given in (**C**,**D**) represent the number of cells of measurements also for (**A**,**B**). * *p* < 0.05 versus Ctr according to *t*-test for two groups.

To prove the role of ROS in the change of the peak I_Na_, hydrogen peroxide (H_2_O_2_), which is the main form of endogenous ROS in cells, was applied. In the donor cells treated with H_2_O_2_, no effects were detected (Figure 8A,C). In SCN10A-hiPSC-CMs, the peak I_Na_ decreased significantly (Figure 8B,D). 

**Figure 9:**
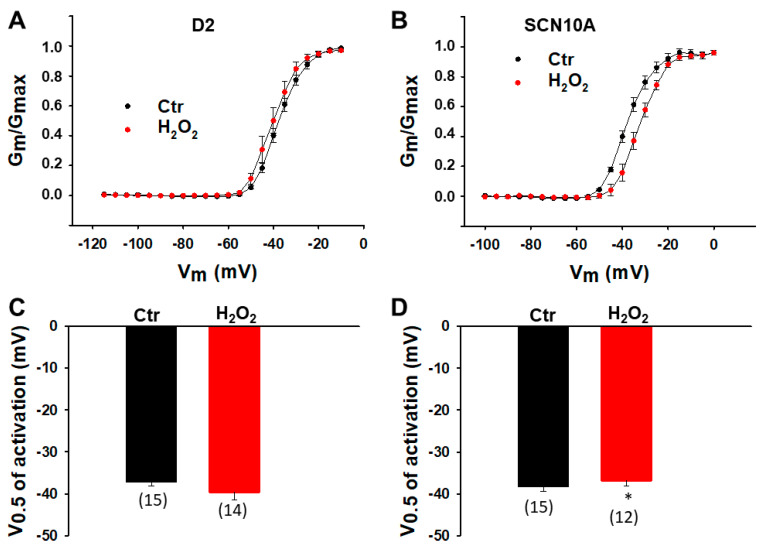
**Effects of H_2_O_2_ on activation of sodium channels in hiPSC-CMs from BrS-patient.** Cells were treated with vehicle (Ctr) or H_2_O_2_ (200 µM) for 2 h. (**A**) Activation curves of peak sodium channel currents (I_Na_) recorded in hiPSC-CMs by the D2 healthy donor. (**B**) Activation curves of peak I_Na_ recorded in SCN10A-hiPSC-CMs. (**C**) Mean values of potential at 50% activation (V0.5) in hiPSC-CMs from D2 healthy donor. (**D**) Mean values of potential at 50% activation (V0.5) in SCN10A-hiPSC-CMs. Numbers given in (**C**,**D**) represent the number of cells of measurements also for (**A**,**B**). * *p* < 0.05 versus Ctr according to *t*-test for two groups.

**Figure 10:**
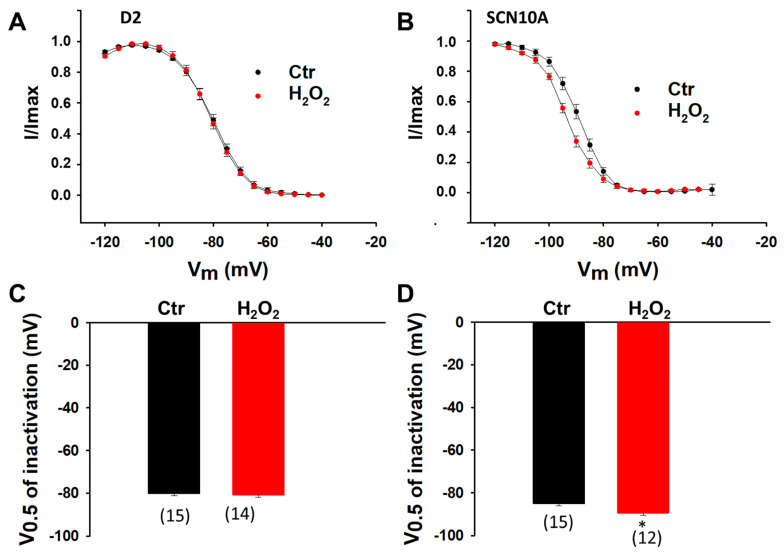
**Effects of H_2_O_2_ on inactivation of sodium channels in hiPSC-CMs from BrS-patient.** Cells were treated with vehicle (Ctr) or H_2_O_2_ (200 µM) for 2 h. (**A**) Inactivation curves of peak sodium channel currents (I_Na_) recorded in hiPSC-CMs from the D2 healthy donor. (**B**) Inactivation curves of peak I_Na_ recorded in SCN10A-hiPSC-CMs. (**C**) Mean values of potential at 50% inactivation (V0.5) in hiPSC-CMs from D2 healthy donor. (**D**) Mean values of potential at 50% inactivation (V0.5) in SCN10A-hiPSC-CMs. Numbers given in (**C**,**D**) represent the number of cells of measurements also for (**A**,**B**). * *p* < 0.05 versus Ctr according to *t*-test for two groups.

**Figure 11:**
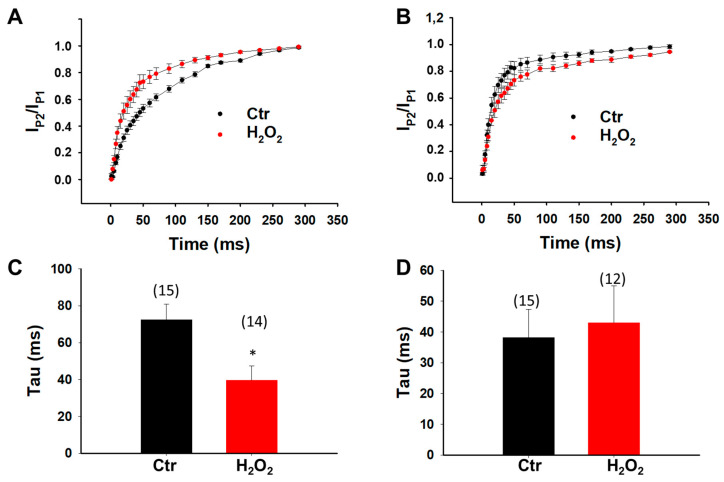
**Effects of H_2_O_2_ on recovery of sodium channels in hiPSC-CMs from donor and BrS-patient.** Cells were treated with vehicle (Ctr) or H_2_O_2_ (200 µM) for 2 h. (**A**) Recovery curves of peak sodium channel currents (I_Na_) recorded in hiPSC-CMs from the D2 healthy donor. (**B**) Recovery curves of peak I_Na_ recorded in SCN10A-hiPSC-CMs. (**C**) Mean values of time constants (Tau) of recovery from inactivation in hiPSC-CMs from D2 healthy donor. (**D**) Mean values of time constants (Tau) of recovery from inactivation in SCN10A-hiPSC-CMs. Numbers given in (**C**,**D**) represent the number of cells of measurements also for (**A**,**B**). * *p* < 0.05 versus Ctr according to *t*-test for two groups.

Since ROS inhibited peak I_Na_, its effects on Na channel gating kinetics were further assessed by treating cells with 200 µM H_2_O_2_ for 2 h. In donor cells, H_2_O_2_ failed to alter the activation and inactivation curves (Figure 9A,C and Figure 10A,C) but accelerated the recovery from inactivation (Figure 11A,C). In SCN10A-hiPSC-CMs, H_2_O_2_ shifted the activation curve to a more positive potential (Figure 9B,D) and the inactivation curve to a more negative potential (Figure 10B,D) without significant influence on the recovery from inactivation (Figure 11B,D). 

#### H_2_O_2_ Effect Was Blocked by a PKC Inhibitor

**Figure 12:**
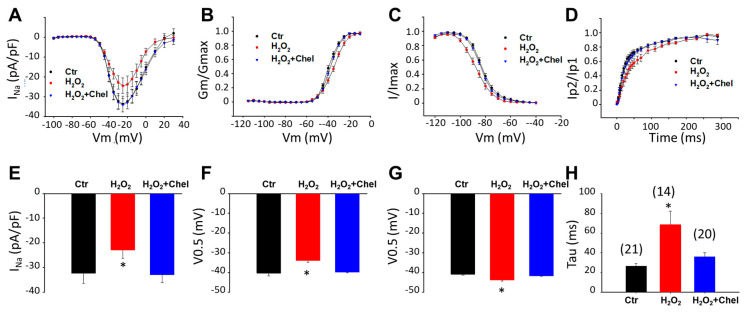
**A PKC inhibitor abolished the effects of H_2_O_2_ on sodium channels in hiPSC-CMs from the BrS-patient.** Cells were treated with vehicle (water and DMSO, Ctr) or H_2_O_2_ (200 µM) or H_2_O_2_ plus 5 µM chelerythrine, a PKC inhibitor (H_2_O_2_ + Chel) for 2 h. (**A**) I–V curves of peak sodium channel currents (I_Na_) recorded in SCN10A-hiPSC-CMs. (**B**) Activation curves of peak I_Na_ recorded in SCN10A-hiPSC-CMs. (**C**) Inactivation curves of peak I_Na_ recorded in SCN10A-hiPSC-CMs. (**D**) Recovery curves of peak I_Na_ recorded in SCN10A-hiPSC-CMs. (**E**) Mean values of peak sodium channel currents at −30 mV in SCN10A-hiPSC-CMs. (**F**) Mean values of V0.5 of activation in SCN10A-hiPSC-CMs. (**G**) Mean values of V0.5 of inactivation in SCN10A-hiPSC-CMs. (**H**) Mean values of time constants (Tau) of recovery from inactivation in SCN10A-hiPSC-CMs. Numbers given in H represent the number of cells of measurements for (**A**–**H**). * *p* < 0.05 versus Ctr according to one-way ANOVA with Holm–Sidak post-test.

To check whether PKC was a downstream factor of ROS in the process of alteration of I_Na_ by LPS, the PKC block chelerythrine was applied. Chelerythrine (5 µM) was applied to SCN10A-hiPSC-CMs for 15 min, and then H_2_O_2_ (200 µM) was added for a further 2 h before cells were recorded. The I–V curve displayed that chelerythrine completely reversed the H_2_O_2_ induced reduction of peak I_Na_ (Figure 12A,E). In addition, chelerythrine also prevented ROS effects on the voltage-dependent activation, inactivation, and time-dependent recovery from inactivation (Figure 12B–D,F–H). These results indicate that PKC is involved in the ROS-induced peak I_Na_ reduction.

## 4. Discussion

Inflammation-induced arrhythmic events have been observed for quite a long time. Around 20 years ago, malignant tachy- and bradyarrhythmia in myocarditis were reported [34]. Inflammation, either acute, or chronic infection or non-infection caused, may influence the process of cardiovascular diseases. Some inflammatory factors, such as endotoxin, cytokines, and C-reactive protein (CRP), can alter the electrophysiology of the cardiomyocytes directly or indirectly [34]. Cytokines are the major modulators of the inflammatory process. Studies show that cytokines were involved in cardiac electrical and structural remodeling and the occurrence of arrhythmias [35]. For instance, TNF-α was shown to enhance arrhythmogenicity in rabbit cardiomyocytes [36]. 

Though increasing evidence shows that inflammation may be involved in the pathogenesis of arrhythmias, few studies assessed the connection between inflammation and BrS. There are some clinical data showing a possible relation between inflammation and BrS, but experimental improvements and mechanistic studies are lacking. This study investigated experimentally the role of the inflammatory response in the pathogenesis of Brugada Syndrome using hiPSC-CMs generated from a Brugada patient and demonstrated, for the first time, that LPS can exacerbate the loss-of-function sodium channel through ROS-PKC signaling. Data from this study may provide new insights into the arrhythmogenesis of BrS.

To study the connection between inflammation and Brs, the first question to be answered in this study is whether inflammation exacerbates the phenotypic changes of BrS. For this purpose, we used LPS to mimic inflammatory responses in healthy and diseased cells. LPS, the endotoxin of Gram-negative bacteria, was usually used to establish inflammatory models in various types of cells, including hiPSC-CMs [28]. Therefore, in this study, we used LPS to challenge hiPSC-CMs from BrS-patients to model the inflammatory responses of the cells and analyze the influences of inflammation on a key phenotypic change, loss-of-function of peak I_Na_, in BrS. 

Our recent study showed that peak I_Na_ was significantly smaller in the SCN10A-hiPSC-CMs than that in healthy donor cell lines, indicating a loss-of-function of sodium channels, which is a key phenotypic change in BrS [7]. The LPS treatment caused a further reduction of peak I_Na_ density in the SCN10A-iPSC-CMs. The reduced peak I_Na_ can reduce the depolarization speed (Vmax) of action potential and, in turn, decelerate the excitation propagation among cardiomyocytes. Since the conduction defect is a typical feature and an arrhythmogenic factor in BrS, this may help to understand how inflammation can contribute to the arrhythmogenesis of BrS. Strikingly, in all the healthy cell lines from three healthy donors, LPS failed to cause a reduction of peak I_Na_. In one healthy donor (D2) cell line, the LPS treatment at higher concentration even increased peak I_Na_ slightly. To understand why LPS did not suppress peak I_Na_ in healthy hiPSC-CMs, we searched available data from the literature. A study in an animal model showed that peak sodium channel current in rat neurons was significantly increased after treatment of LPS [37], which was observed in our study in D2 cells with a high concentration of LPS. However, two studies assessing cardiac sodium channels of rabbits showed that LPS failed to change the peak I_Na_ [38,39], consistent with our observation in healthy hiPSC-CMs. In a rat model of sepsis, papillary muscles showed a decreased action potential (AP) magnitude and the rate of depolarization. When tetrodotoxin (TTX) was utilized to block the sodium channel, similar changes in APs were detected. It was concluded that the sepsis could suppress sodium channel current [40], which is consistent with our result in BrS-hiPSC-CMs treated by LPS. Taken all together, previous studies detected different effects of LPS on peak I_Na_, increase, decrease, or no effect. These data imply that the LPS effects on sodium channels can be influenced by extra factors. The difference between healthy and BrS cells regarding the effects of LPS on peak I_Na_ suggests that the variant in SCN10A rendered sodium channels to be inhibited by LPS. How the variant in SCN10A renders sodium channels to be sensitive to LPS is still an open question and needs to be clarified in future studies.

The next question is how LPS inhibited peak I_Na_ in BrS cells. It is known that the amplitude of peak I_Na_ is determined by the channel open activity and channel expression level in the cell membrane. In this study, we focused on the influence of LPS on channel open activity. For this purpose, we analyzed the channel gating kinetics, including activation, inactivation, and recovery from inactivation of peak I_Na_ in cells challenged by LPS. 

The reduction of peak I_Na_ in SCN10A-cells can be explained by the suppression of activation, enhancement of inactivation, and deceleration of recovery. The novel data showing that LPS induced a reduction of peak I_Na_ density in BrS cells demonstrates that the endotoxin or some inflammatory factors may be able to unmask the BrS-phenotype and exacerbate arrhythmias in some BrS-patients.

Another important point is how LPS changed sodium channel currents and gating kinetics. It is well-known that reactive oxygen species (ROS) play an important role in cell signaling. ROS is involved in the different pathology processes, including inflammation, neurodegeneration, diabetes, atherosclerosis, and aging. The phenomenon that LPS can increase ROS generation in cardiomyocytes has been reported [41,42] and is in agreement with the result of the current study. ROS may also participate in the process of ion channel regulation via direct or indirect mechanisms. In an experiment on rat neurons, Wang et al. observed that endogenous ROS increased peak sodium current in muscle afferent DRG neurons in an unknown way [29]. This is in contrast to our data in the current study. However, Liu et al. used NADH and other drugs to enhance ROS generation in HEK cells and cardiomyocytes, and they detected an increase in ROS production and a reduction of peak I_Na_ in both types of cells [30], suggesting that ROS may reduce peak I_Na_, in agreement with our data in BrS-hiPSC-CMs. To examine the role of ROS in the LPS-induced reduction of I_Na_ in the BrS-hiPSC-CMs, we first investigated the effects of the ROS scavenger NAC (*N*-acetyl-l-cysteine). NAC significantly reduced the LPS effects on the sodium channels, including channel currents and gating kinetics. NAC alone showed no effect on sodium current, suggesting that ROS was involved in LPS effects. ROS generation was also observed in our hiPSC-CMs treated with LPS. DCF fluorescence assay showed that LPS induced a significant increase in ROS generation in iPSC-CMs, the second evidence for the involvement of ROS in the LPS effects. Of note, LPS induced similarly the ROS generation in both donor and BrS cells. This raised a question: why did LPS not inhibit I_Na_ in donor-hiPSC-CMs? Therefore, we further assessed the effects of H_2_O_2_, the main form of ROS in cells, on sodium channel currents and kinetics in donor- (D2) and SCN10A-hiPSC-CMs. Indeed, H_2_O_2_ mimicked the effects of LPS on sodium channel currents and gating kinetics in BrS cells, but not in D2 cells. These results confirmed that ROS contributed to the LPS-induced reduction of sodium current in the SCN10A-hiPSC-CMs and indicated that the ROS effects on sodium channels are gene variant related. Probably, the change of nucleoside leads to structural or functional changes in channel proteins and, in turn, renders the channel sensitive to ROS. 

Aside from inflammation, ROS signaling can participate in the processes of many other diseases, such as stress, ischemia, diabetes, cancer, etc. Co-existence of BrS and cancer has been reported [43,44,45], but the concrete relation is unclear. The Brugada pattern has also been previously reported in the setting of fever, diabetes, stress, ischemia, and myocardial and pericardial diseases [46,47,48,49], all of which may contain pathogenic roles of ROS. ROS signaling might be involved in the pathogenesis of BrS in those diseases. In different diseases, however, the BrS phenotype can be evoked by different signaling.

Next, we tried to unveil the mechanism behind the ROS effect on sodium channels. PKC was shown to participate in LPS-induced ROS generation. A recent study reported that the genetic deletion of PKC delta in mice prevented an increase in total or mitochondrial ROS in cardiomyocytes after LPS exposure [50]. This result is in agreement with another study by Liu et al. [30]. In our current study, we used H_2_O_2_ to mimic the ROS effects on sodium channels. The application of a PKC inhibitor, chelerythrine [51], was able to completely prevent the H_2_O_2_ effects. This finding indicates that PKC is probably a downstream factor of ROS in cardiomyocytes. 

PKC was shown to be activated by H_2_O_2_ [52]. It was also shown that PKC inhibitor could suppress the effect of H_2_O_2_ on late sodium current and action potential in cardiomyocytes, suggesting that PKC is an important factor in ROS signaling [53]. Qu described that the phosphorylation of S1505 in cardiac sodium channels by PKC altered the steady-state inactivation and the effect was completely abolished by the mutation S1505A. These data indicate that amino acid S1505 in the cardiac sodium channel is critical for channel regulation by PKC [54]. Our study demonstrated that H_2_O_2_ reduced peak I_Na_ and shifted the inactivation curve of I_Na_ to more negative potential in SCN10A-hiPSC-CMs and both effects were suppressed by the PKC inhibitor. This result implies that PKC mediated the effect of H_2_O_2_ on peak I_Na_ in SCN10A-hiPSC-CMs. 

Taking all the data together, LPS may increase the risk of arrhythmias in BrS-patients with the SCNB10A mutation by exacerbating loss-of-function of sodium channels by enhancing ROS-PKC signaling, which can suppress peak sodium channel current via changing the channel gating kinetics. 

## 5. Conclusions

This study demonstrated that LPS can exacerbate the loss-of-function of sodium channels by suppressing peak sodium channel currents and changing sodium channel gating kinetics. Inflammation may play an important role in the pathogenesis of some BrS-patients. Anti-inflammation treatment may be helpful in preventing the occurrence of arrhythmias in BrS-patients. 

## 6. Study Limitations

Our study detected different LPS effects on the healthy donor iPSC-CMs and the BrS iPSC-CMs. The exact reason for the difference is unclear. We speculate that it may be related to the patient-specific gene variants or mutations. In this study, we used healthy donor iPSC-CMs as the control group, but an isogenic control using the CRISPR/Cas9-mediated genome editing was lacking. 

The study exhibited that PKC was involved in LPS and ROS effects on I_Na_ in BrS-hiPSC-CMs, but which subtype of PKC exerted the effect was not addressed.

One of the main limitations of iPS-CMs as a model for arrhythmogenic diseases is the immature phenotype of hiPSC-CMs. The differences in cell properties, including electrical activities between hiPSC-CMs and native cardiomyocytes, should be also considered in interpreting the data of this study.

## Data Availability

All the data reported in this study are contained in the paper.
